# Neuromodulation Using Electroosmosis

**DOI:** 10.1088/1741-2552/ac00d3

**Published:** 2021-06-02

**Authors:** Sai Siva Kare, Corey M Rountree, John B Troy, John D Finan, Laxman Saggere

**Affiliations:** 1 Department of Mechanical and Industrial Engineering, University of Illinois at Chicago, Chicago, Illinois, USA; 2 Department of Biomedical Engineering, Northwestern University, Evanston, Illinois, USA

**Keywords:** Chemical retinal stimulation, pneumatic actuation, electroosmotic flow, photoreceptor degenerative diseases, retinal prostheses, neural interface

## Abstract

**Objective.:**

Our laboratory has proposed chemical stimulation of retinal neurons using exogenous glutamate as a biomimetic strategy for treating vision loss caused by photoreceptor degenerative diseases. Although our previous in-vitro studies using pneumatic actuation indicate that chemical retinal stimulation is achievable, an actuation technology that is amenable to microfabrication, as needed for an in-vivo implantable device, has yet to be realized. In this study, we sought to evaluate electroosmotic flow (EOF) as a mechanism for delivering small quantities of glutamate to the retina. EOF has great potential for miniaturization.

**Approach.:**

An EOF device to dispense small quantities of glutamate was constructed and its ability to drive retinal output tested in an in-vitro preparation of photoreceptor degenerate rat retina.

**Main Results.:**

We built and tested an EOF microfluidic system, with 3D printed and off-the-shelf components, capable of injecting small volumes of glutamate in a pulsatile fashion when a low voltage control signal was applied. With this device, we produced excitatory and inhibitory spike rate responses in photoreceptor degenerate rat retinae. Glutamate evoked spike rate responses were also observed to be voltage-dependent and localized to the site of injection.

**Significance.:**

The EOF device performed similarly to a previously tested conventional pneumatic microinjector as a means of chemically stimulating the retina while eliminating the moving plunger of the pneumatic microinjector that would be difficult to miniaturize and parallelize. Although not implantable, the prototype device presented here as a proof of concept indicates that a retinal prosthetic based on EOF-driven chemical stimulation is a viable and worthwhile goal. EOF should have similar advantages for controlled dispensing of charged neurochemicals at any neural interface.

## 1. Introduction

A progressive loss of retinal photoreceptor (PR) cells due to such incurable diseases as retinitis pigmentosa and macular degeneration leads to permanent loss of vision [1,2]. To treat such diseases, decades of ongoing research and development in the field of retinal-based prostheses have demonstrated partial vision restoration by artificially stimulating surviving neurons using several neuromodulation technologies [3–8]. These technologies differ fundamentally in the principle used (e.g. ultrasonic [4], mechanical [5], optogenetic [9,10], electrical [6] or chemical [7, 8]) for stimulating surviving retinal neurons to generate visual function. Among these technologies, electrical retinal stimulation is the most widely studied and remains the only clinically approved method for functional vision restoration in humans [11–14]. Chemical stimulation is attractive because it evokes neural responses by injecting exogenous neurotransmitters that activate receptors on the target neurons, mimicking natural synaptic action [15,16]. Recent in-vitro studies involving chemical stimulation of retinal neurons, using the primary neurotransmitter glutamate, have shown the feasibility of achieving both epiretinal [7,8] and subretinal [17] stimulation of PR degenerated rat retinae. In addition to single site retinal stimulation using micropipettes, researchers have demonstrated subretinal multisite, patterned stimulation using a microfluidic prototype synapse chip [18] consisting of an array of microscopic pores. One commonality shared between these studies involving micropipettes and the multiport microfluidic device is the use of an external pressure injector system to drive the glutamate solution by controlling the volume, pressure and frequency of pulsatile glutamate injections. Although convenient for fundamental studies, pressure injection cannot be adapted easily for an implantable chemical-based retinal prosthesis. Therefore, it is crucial to find an alternate fluid actuation mechanism that permits miniaturization of the system for retinal implantation, while maintaining glutamate flow characteristics suitable to restore visual function.

Widely used in microfluidic devices as an actuation mechanism, electroosmosis is one candidate that well suits the requirements of a subretinally implantable chemical synapse chip. Generally, in electroosmotic flow (EOF), bulk fluid motion is generated by applying an electric field across a channel filled with an ionic liquid. One of the major advantages of electroosmosis is the complete elimination of any mechanical moving part (such as active microvalves [19], pumping diaphragm or membrane [20,21]) in the microfluidic system, improving the device simplicity and reliability, which is a significant requirement of an implantable device. Several microfluidic systems employing EOF have been developed with different channel dimensions varying from hundreds of micrometers to a few nanometers [22–24], allowing dense arrays of microchannels that could be independently addressable by applying an electric field across selected microchannels. Studies have shown that EOF can be generated at lower voltages by reducing the channel length and separation between electrodes supplying the electric field [25–27], which is encouraging for an implant consisting of microfabricated channels and electrodes. By patterning the electrodes around a microchannel using microfabrication, EOF can be generated and controlled in a compact form [28,29]. Also, given the ionic nature of neurotransmitter chemicals such as glutamate, EOF could act as a suitable driver for a neurotransmitter delivery system [12, 20].

**Figure 1:**
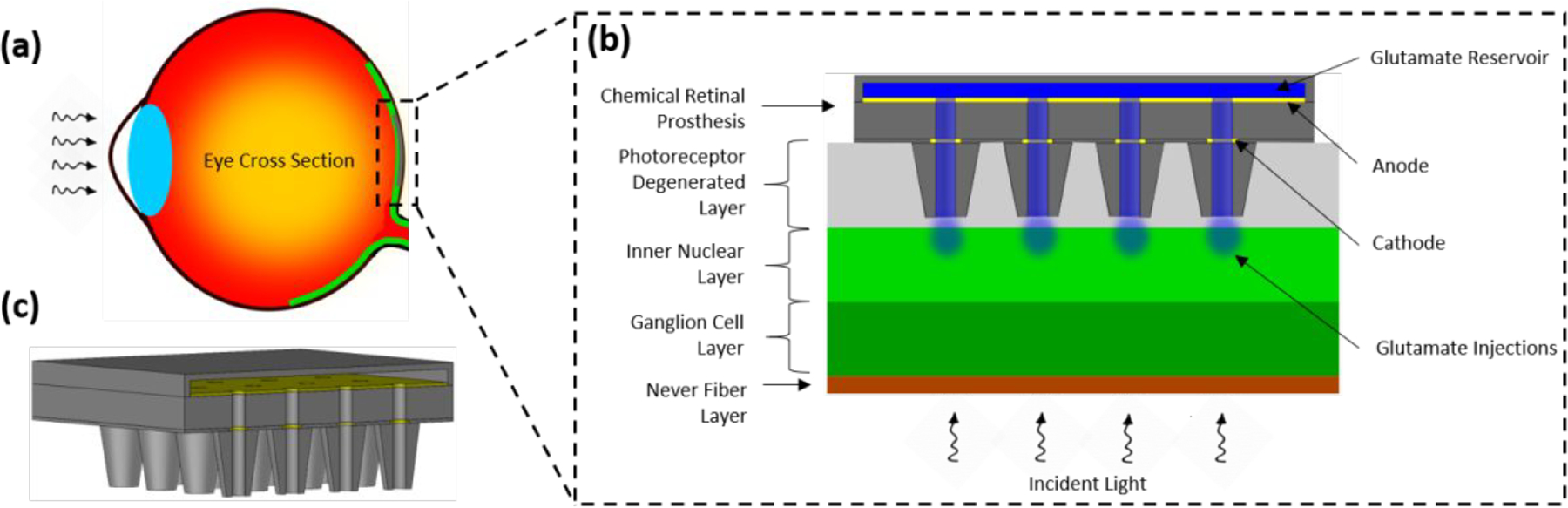
A conceptual design of an implantable EOF microfluidic device for delivering localized and minute glutamate puffs near the functional layers of a photodegenerated retina. (a) Schematic showing the placement of the subretinal chemical stimulator within the eye. (b) A sectional view of the device-retina interface showing a rudimentary EOF device (grey) with porous structure for generating EOF and individually addressable electrodes for injecting glutamate (blue). By generating a voltage difference between anode and cathode an electric field will be set-up across the porous structure, thus, releasing glutamate injections in response to incident light. For simplicity, the retina’s inner plexiform layer is not indicated. (c) Cross sectional view of the EOF device showing an array of microneedles necessary for generating patterns on the retina to restore visual function and an onboard reservoir for glutamate storage.

Fig. 1 shows a conceptual EOF device that can be interfaced with a PR degenerated retina to inject small puffs of glutamate in response to incoming light, thus generating an artificially evoked visual pattern. An onboard reservoir replenishes spent glutamate and electrodes patterned around individual glutamate outlets generate electric fields to inject glutamate onto retinal neurons where the missing photoreceptors would have done so. Although, EOF provides several proven advantages in the microfluidic realm, an in-depth understanding of EOF of the neurotransmitter glutamate and its implementation for in-vitro chemical stimulation of retinal neurons has never been explored. Herein we demonstrate that EOF can deliver glutamate onto retinal neurons with spatial and temporal characteristics required for visual restoration by this application, eliciting neural responses in-vitro at voltages as low as 2 V. Using a scaled-up version of the EOF system described in the conceptual design, we characterized the prototype device for controlled delivery of 1 mM glutamate and fine-tuned the flow rate and pulsatile injection parameters to those needed for in-vitro retinal stimulation. These results constitute a step forward for the eventual implementation of chemical neuromodulation in retinal prosthetics and other applications because they prove the feasibility, for the first time, of a fluid actuation approach that is easier to miniaturize than other fluid actuation approaches.

## 2. Materials and Methods

### 2.1 Fabrication and EOF Design

**Figure 2:**
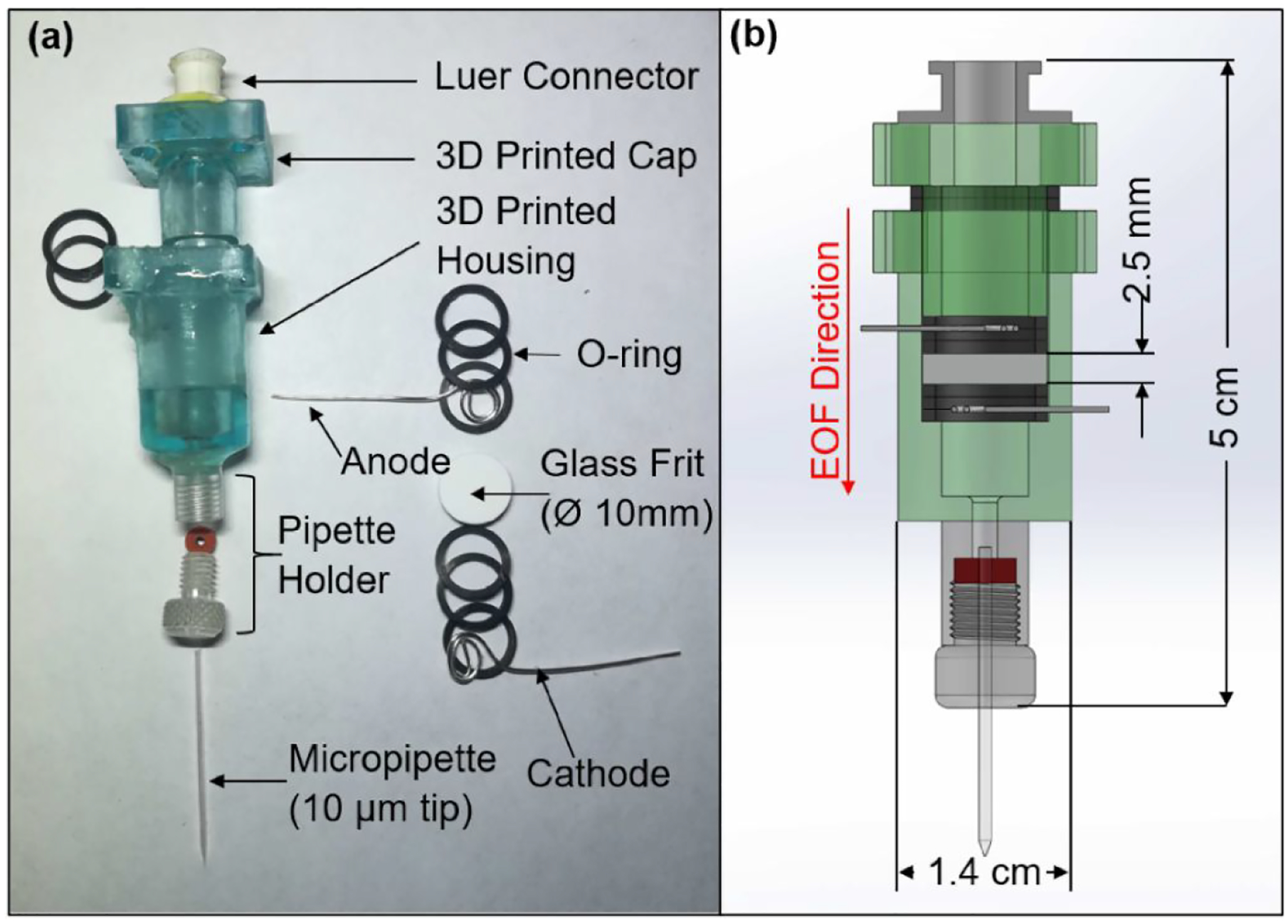
Disassembled components and assembled view of the EOF device used for chemical stimulation of rat retinae. (a) The device included a capped 3D printed housing, a micropipette, a glass frit, O-rings and silver electrodes. The glass frit was used to generate EOF of glutamate and voltage was applied between silver electrodes serving as anode and cathode. The micropipette was secured to the housing by a section of standard pipette holder. Glutamate loading was accomplished using a female Luer connector epoxied at the top of the 3D printed cap. Prior to the experimental trials, the porous glass frit was soaked in 1 mM glutamate solution to ensure that no air bubbles were trapped inside its pores. (b) A cross sectional diagram of the device with overall dimensions, showing the order in which the parts were assembled.

To investigate the proof of concept of utilizing EOF to chemically stimulate retina in-vitro, previously established design criteria [16,17] were considered, where a standard micropipette (10 μm tip diameter, World Precision Instruments, Sarasota, FL, USA) was used to inject glutamate solution pneumatically via a programmable pressure injector. Here, we replaced the pneumatic actuation system with an electroosmotic device that could accept a standard micropipette and inject glutamate upon controlled voltage application. Fig. 2 shows various parts (Fig. 2a) of the entire device used for stimulation trials and the cross-sectional view (Fig. 2b) of the 3D printed cap and a housing containing two silver electrodes (99.9 % Ag, 0.5 mm diameter, Sigma Aldrich, St. Louis, MO, USA), O-rings, and a glass fritted disc (10 mm diameter, 2.5 mm thick and pore size between 4 and 5.5 μm, Chemglass Life Sciences, Vineland, NJ, USA) known for high EOF rate generation at low voltages [22,27]. O-rings (1 mm wide, 8 mm inner diameter, Square-Profile Oil-Resistant Buna-N O-Ring, McMaster-Carr, Elmhurst, IL, USA) were used to secure the frit and prevent glutamate leakage from the 3D printed parts (50 μm layer thickness, EPAX X1 UV LCD 3D Printer). To connect the micropipette to the 3D printed housing, a 1 cm section of a standard pipette holder (QSW-A10P, Warner Instruments, Hamden, CT, USA) was cut and glued to the housing using waterproof two-part clear epoxy (3M ScotchWeld DP420 adhesive). A female Luer connector epoxied to the cap enabled filling of the device with 1 mM glutamate solution, which was prepared by mixing L-glutamic acid (Sigma-Aldrich, St. Louis, MO, USA) in de-ionized (DI) water. We used a glutamate concentration (1 mM) found to be effective at retinal stimulation in a previous study [8,16]. By applying a pulsating voltage of a set frequency across the glass frit through the silver electrodes (separated by 5 mm), EOF of glutamate solution was generated and injected into the retina through the micropipette.

### 2.2 Characterization of EOF Flow Rate

**Figure 3:**
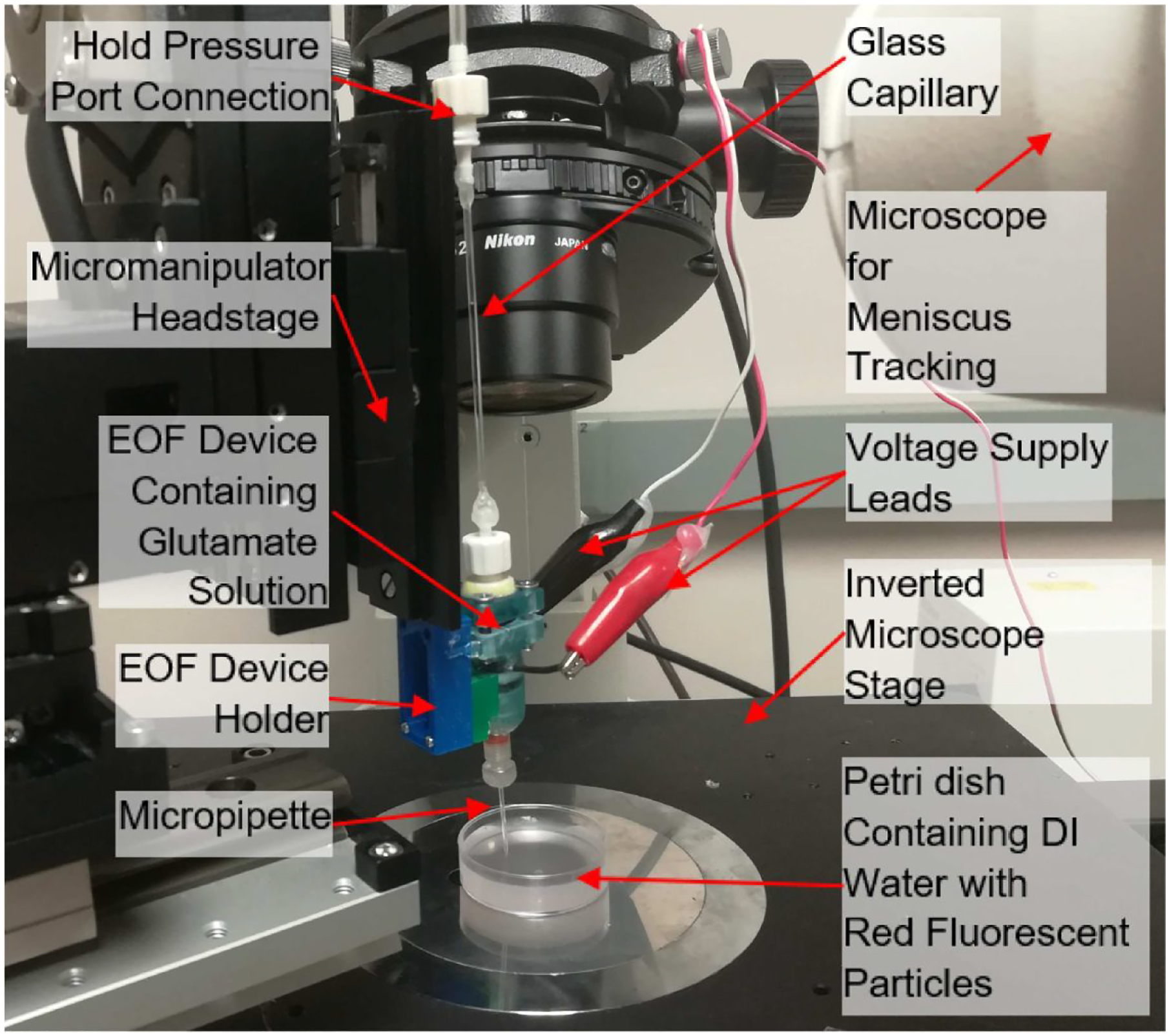
Set-up for measuring the injection volume of 1 mM glutamate against applied voltage pulses. A capillary attached to the top of the device was used to visualize displacement of the meniscus before and after glutamate injection for different voltages. A digital camera attached to a stereo microscope was used to image and track the meniscus. After lowering the micropipette tip into a Petri dish using a micromanipulator, a hold pressure was applied to prevent glutamate leakage through the micropipette tip. Once no leakage (meniscus movement) had been confirmed in the camera’s live feed, the device was supplied with voltage pulses. Voltage induced release of glutamate was confirmed by imaging the micropipette tip using epifluorescence microscopy (connected to an inverted microscope). Fluorescent particles dispersed in DI water flowed from the tip in concert with the applied voltage pulses.

To ensure that our 3D printed EOF device could dispense glutamate at low concentration [18] in a controlled manner, we measured the volume of 1 mM glutamate solution released into solution when a pulsating actuation voltage was applied. Dispensed glutamate was measured by precisely tracking the displacement of the meniscus in a transparent conduit. A glass capillary (internal/outer diameter: 1.16/1.5 mm, World Precision Instruments, Sarasota, FL, USA) was filled with 1 mM glutamate solution through the device’s Luer connector (Fig. 2). Then, the device and capillary assembly were mounted vertically on a custom 3D printed holder attached to the headstage of a three-axis micromanipulator (MP-285, Sutter Instruments, Novato, CA, USA) as shown in Fig. 3. Finally, the device tip was lowered into a Petri dish containing DI water laced with red fluorescent microparticles (1 μm diameter, red fluorescent polystyrene microspheres, ThermoFisher Scientific, Waltham, MA, USA). To visualize and confirm pulsating glutamate flow through the micropipette tip, fluorescent particle movement near the micropipette tip was monitored using an epifluorescence imaging system (Andor Zyla 5.5 sCMOS camera and Nikon Intensilight C-HGFI epifluorescence illuminator, Tokyo, Japan) connected to an inverted microscope (Nikon Eclipse Ti-E inverted microscope, Nikon, Tokyo, Japan). The narrow opening of the pMEA chamber (internal diameter: 26 mm) and restricted maneuvering space for the EOF device necessitated a vertical orientation. Consequently, a pressure injector system (Pressure Injector System PM-8, Harvard Apparatus, Holliston, MA, USA) was used to apply a hold pressure (suction) to prevent glutamate leakage from the micropipette tip due to the fluid pressure head (height of the glutamate column inside the device). Such leakage (and consequently the need for negative pressure) would be reduced substantially for an in-vivo implantable device where a far smaller pressure head would be present.

To inject the glutamate solution in a pulsatile manner, square voltage pulses were applied with amplitudes of 2, 5, and 10 V (corresponding to an electric field of 400, 1000 and 2000 V/m respectively). A sourcemeter (Keithley 2611A Sourcemeter, Keithley, Cleveland, OH, USA) was used to trigger 100 ms voltage pulses at 1 Hz frequency for 100 seconds via a custom script code in Labview (National Instruments, Austin, TX, USA). Since the inner diameter of the glass capillary is fixed, the total volume dispensed by the device was determined by measuring the meniscus displacement before and after 100 s. Images of meniscus displacement were captured using a 5 MP digital microscope camera (Moticam 5, Motic, Kowloon, Hong Kong) attached to a stereo microscope (SMZ 745T, Nikon, Tokyo, Japan) mounted on a boom stand and measured using image analysis software (Fiji, Schindelin et. al. [30]). A measurement of meniscus displacement was taken for each voltage trial to control for evaporation or leakage of glutamate. Then 100 cycles of 100 ms voltage pulses (for 2, 5, and 10 V) were applied and the corresponding meniscus displacement measured. The control measurement of meniscus displacement described above was subtracted from these measured meniscus displacements when voltage pulses were applied to arrive at the measures recorded of volumes injected.

### 2.3 Characterization of EOF Switching Time

An important requirement of the actuation mechanism intended for retinal stimulation is the ability to switch glutamate injections ON or OFF with minimum latency. The capacity to do this sets a limit for the highest frequency of glutamate stimulation [8]. To verify a rapid ON-OFF injection response time from the EOF device for 100 ms injection pulses, fluorescence microscopy was utilized with an identical experimental setup as described for the flow rate characterization section (and Fig. 3). To visualize the glutamate boluses ejected from the micropipette tip clearly, the device was tilted 20° from vertical. To render the injection pulses near the micropipette tip visible the 3D printed housing was filled with 1 mM glutamate, while the micropipette was filled with 0.08% (w/w%) fluorescein – DI water solution. This ensured that EOF is generated strictly due to 1 mM glutamate instead of the fluorescein solution. Injections, with 100 ms pulse width and 2, 5, and 10 V actuation voltage, were triggered at 1 Hz frequency using the sourcemeter and the resulting fluorescein injections into a Petri dish containing clear DI water and were captured at 100 frames per second (FPS) using the epifluorescence imaging system connected to the inverted microscope.

### 2.4 Retinal sample preparation

Transgenic non-pigmented homozygous S334ter-3 rats (LaVail Laboratory, University of San Francisco, CA) were mated with pigmented Hooded Long Evans rats (Charles River Laboratories, Wilmington, MA) to produce pigmented, hemizygous offspring expressing a single copy of the S334ter-3 transgene that has been well-characterized to generate photoreceptor degeneration that is similar to the human condition [31–33]. Retinal explants were taken from a total of 6 completely photoreceptor-degenerated, hemizygous S334ter-3 rats (postnatal day 130–150, either male or female) following euthanasia by carbon dioxide and cervical dislocation. Explanted retinae were placed onto a perforated multielectrode array (pMEA, pMEA200/30iR-Ti, Multichannel Systems, GmbH) with ganglion cell side towards the electrodes and perfused with room temperature (22°C) Ames medium (flow rate 3 mL/min) oxygenated with carboxy gas (95% oxygen and 5% carbon dioxide) for at least 30 minutes prior to pMEA recordings. The retina was perfused from both the top through the pMEA perfusion chamber and the bottom through the perforations on the pMEA to produce adequate perfusion. To ensure firm contact between the pMEA electrodes and retinal tissue throughout the entire duration of the experiment, slight suction was applied to the bottom of the pMEA. This necessitated removal of the standard pMEA heating plate, and in-line heaters were not used to preclude noise in pMEA recordings arising from bubble generated during heating which prevents smooth suctioning. Consequently, all experiments were conducted at room temperature.

All animal experiments were conducted in accordance with the guidelines outlined by the National Research Council’s Guide for the Care and Use of Laboratory Animals. All experimental protocols were reviewed and approved by the Institutional Animal Care and Use Committee of the University of Illinois at Chicago.

### 2.5 Experimental setup

**Figure 4:**
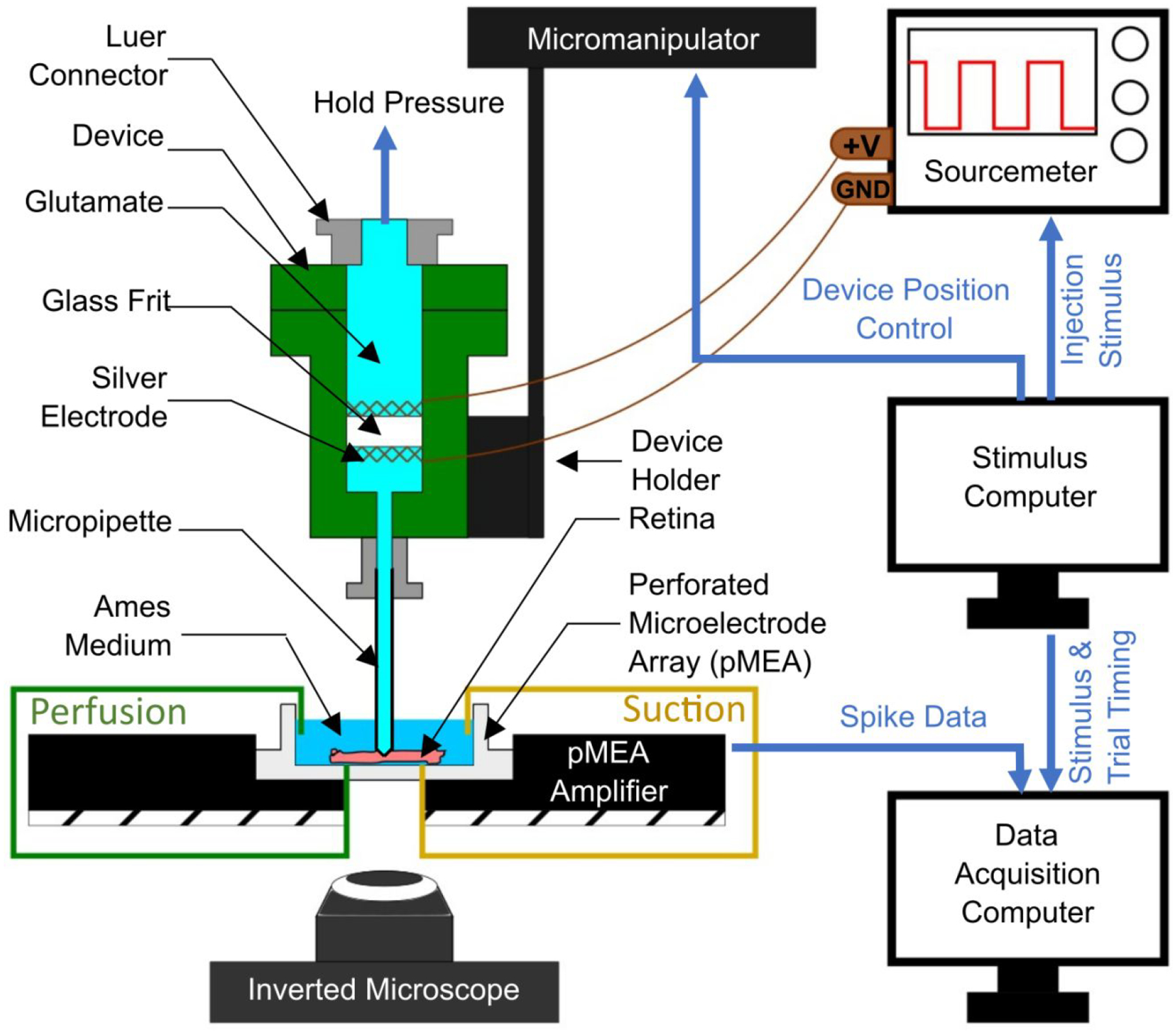
A schematic showing the experimental set-up used for electroosmotic stimulation of the retina in-vitro. An explanted rat retina was placed onto a perforated microelectrode array pMEA), with the retinal ganglion cells (RGCs) contacting the electrodes of the pMEA. The retina’s health was sustained by perfusing it with oxygenated Ames medium, while maintaining perfusion from both top and bottom of the pMEA chamber. The glutamate filled electroosmotic flow (EOF) device was positioned using a micromanipulator (controlled by a stimulus computer) and the micropipette was interfaced with the retina while observing the tip-retina contact through an inverted microscope. When the contact was visually confirmed, the tip was lowered 40 μm further into the retina. Subsequently, retinal stimulations were achieved by EOF actuation of glutamate, generated by supplying voltage across the glass frit by two silver electrodes. The anode is connected to the positive voltage terminal on the sourcemeter, while the cathode is grounded. The stimulus computer was used to generate timed voltage signals with a set frequency, amplitude and number of stimuli. RGC spike data were obtained via the pMEA and the stimulus timings were recorded by a data acquisition computer.

The pMEA recording apparatus (MEA1060, Multichannel Systems, GmbH) was placed on top of an inverted microscope (Nikon Eclipse Ti-E, Tokyo, Japan) to simultaneously allow neural recordings and visual observation of the retina (Fig. 4). The pMEA used for retinal recording featured 60 electrodes of 30 μm-diameter that were located at the intersections of a square grid laid out in an (8×8) pattern with an inter-electrode spacing of 200 μm, except at its four corners. The retinal ganglion cell (RGC) response signals picked up by the 60 electrodes of the pMEA were amplified and acquired into a computer through the pMEA system with MC Rack software (MEA1060, Multichannel Systems, GmbH). After identifying pMEA electrodes with robust neural activity, the EOF device was positioned to enable stimulation over one of those electrodes using a three-axis, motorized, precision manipulator (MP-285, Sutter Instruments, Novato, CA) with sub-micron positioning accuracy.

The glass tip of the EOF device was positioned directly over targeted electrodes and then lowered until it was 40 μm below the retinal surface (to target the outer plexiform layer of the retina [17]), which was detected through visual observation. Once in position, 30 trials of EOF injections were initiated with a time-width of 100 ms and EOF voltages of 2, 5, and 10 V. To achieve repeatable, high accuracy spatiotemporal modulation, all instruments used for EOF chemical stimulation were computer controlled via a digital-to-analog DAQ board (PCI-6251, 16-bit, National Instruments, Austin, TX) and custom programs coded in LabView (National Instruments, Austin, TX).

### 2.6 Neural response data acquisition and analysis

Neural signals in the form of RGC spikes were recorded with the pMEA system using MC Rack software at a 10 kHz sampling rate with a high-pass filter (200 Hz cutoff) and an amplitude threshold of approximately −16 μV. Spikes were sorted using the wavelet clustering package, Wave clus, developed by Quiroga et al [26] in MATLAB. Following spike sorting, the spike rate of each unit was estimated by time binning the spikes with a binwidth of 25 ms producing average prestimulus time histograms (PSTHs) using custom MATLAB code. Responsive units were identified using a fano factor derived response variable (see [16] for details) and further filtered by excluding units with spike rate response frequencies less than 3 Hz to eliminate neural responses with low signal-to-noise ratios. Each responsive PSTH was further analyzed to extract the amplitude, time-width, and latency of the spike rate responses. The spike rate amplitude was calculated as the difference between the extrema and mean spike rate, with negative and positive amplitudes characterizing inhibitory and excitatory phases, respectively. The temporal characteristics of spike rate responses were estimated by measuring the time-width (full-width at half maximum) and the latency, which was defined as the time difference between the injection and the peak response. The effects of EOF voltage on these spike rate parameters were analyzed using a non-parametric two-sided Mann-Whitney U test because the data were not normally distributed.

The spatial localization of EOF responses was investigated by mapping the locations of glutamate-responsive somal spike shapes [41] for EOF at 2 V using the distance vectors separating the EOF injection site from the electrodes with glutamate responses. Units with somal, as opposed to axonal, spike shapes were used because somata are colocalized with the sites of the recording electrodes while axonal spikes are not. The spatial distribution was mapped by assembling these distance vectors into a 2D histogram via spatial binning to represent the approximate locations of electrodes where responsive RGCs were recorded. This histogram was then fitted with a two-dimensional Gaussian spread function that had parameters as specified in equation 1, where x, y and w are in μm.

(1) $$\text{spread}\left(\text{x},\text{y}\right)=\text{a.exp}\left(-\left(\frac{{\text{x}}^{2}+{\text{y}}^{2}}{2{\text{w}}^{2}}\right)\right)$$

Coefficients (with 95% confidence bounds):

a = 5 (4.9, 5.1)

w = 157.3 (155.2, 159.3)

## 3. Results

In this work, we have characterized the potential of localized stimulation of retinal neurons using EOF to inject small boluses of the neurotransmitter glutamate into the subretina of photoreceptor-degenerated rat retinas. To accomplish this goal, we first tuned the glutamate EOF properties by characterizing the flow rate and EOF switching time without any resistance at the tip of the micropipette (i.e. absent the rat retina). This ensured that the device was capable of injecting sufficient glutamate at temporal frequencies found effective in previous chemical stimulation experiments [7,8,16]. Subsequently, the spike rate responses of retinal ganglion cells (RGCs) to EOF glutamate stimulation were examined to determine the population level response to glutamate. Next, we investigated how different EOF voltages affected the spike rate parameters (e.g. amplitude, latency, width) of glutamate-evoked spike rate responses. Finally, we examined the spatial spread of retinal responses elicited through EOF glutamate stimulation.

### 3.1 Voltage versus glutamate EOF rate

**Figure 5:**
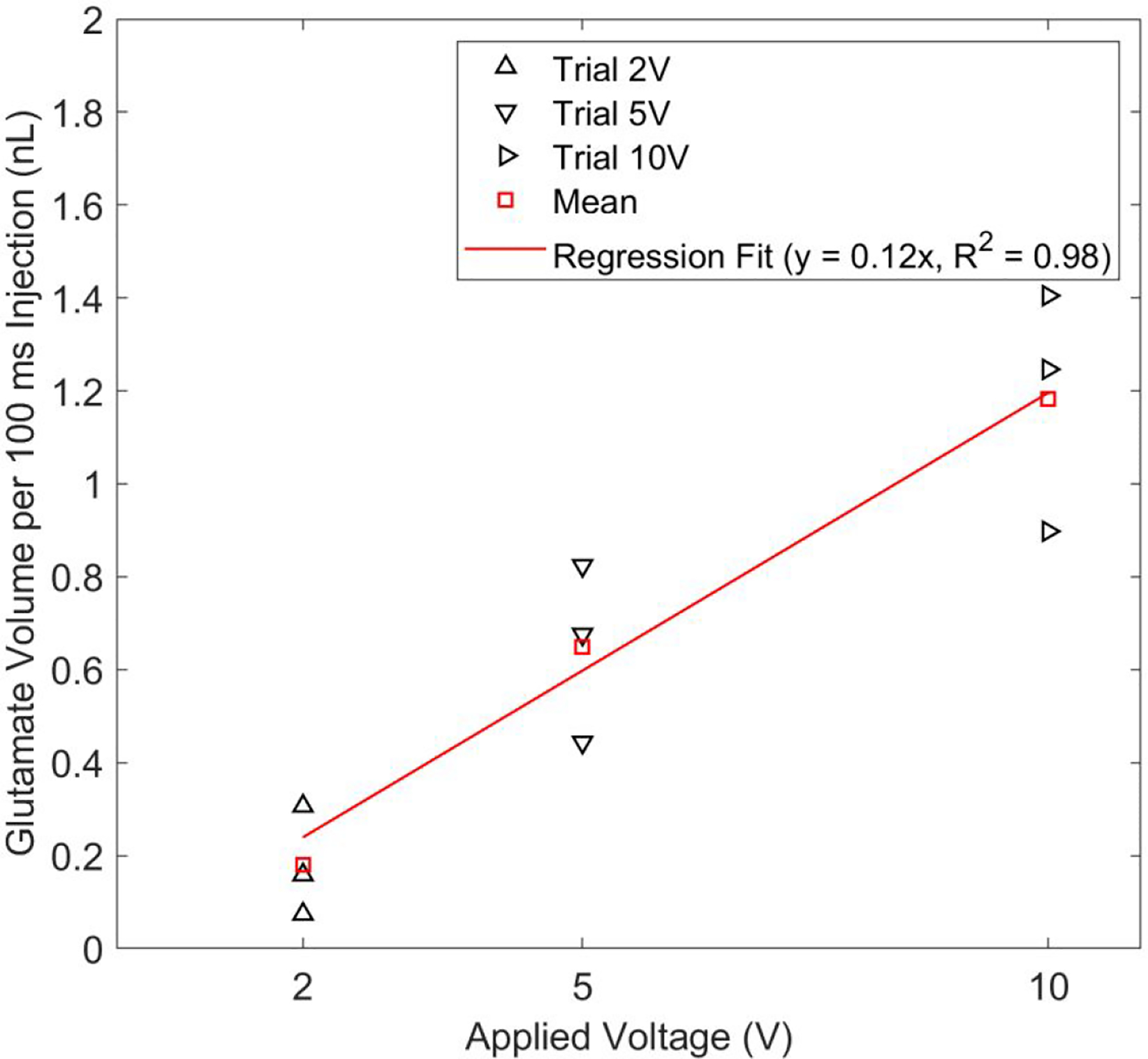
Glutamate volume per 100 ms injection (nL) versus applied voltage (V) characteristic of the EOF device found using the meniscus tracking method. Voltage pulses were applied for 100 cycles with a time-width of 100 ms at 1 Hz frequency. The plot obtained from three trials for each actuation voltage shows that higher glutamate injection volumes can be achieved by increasing the voltage amplitude.

To maintain low glutamate injection volume for in-vitro stimulation we studied the voltage versus glutamate flow characteristics of the EOF device within a voltage range of 2 to 10 V. Glutamate EOF rate estimation using a meniscus tracking method revealed that the glutamate volume injected per 100 ms increased with voltage. Within the limits of the pixel resolution of the acquired meniscus images, the lowest (2 V actuation pulse) and highest (10 V actuation pulse) glutamate volume injected was measured to be approximately 0.2 and 1.2 nL per 100 ms injection (Fig. 5) respectively.

### 3.2 Glutamate EOF onset latency

**Figure 6:**
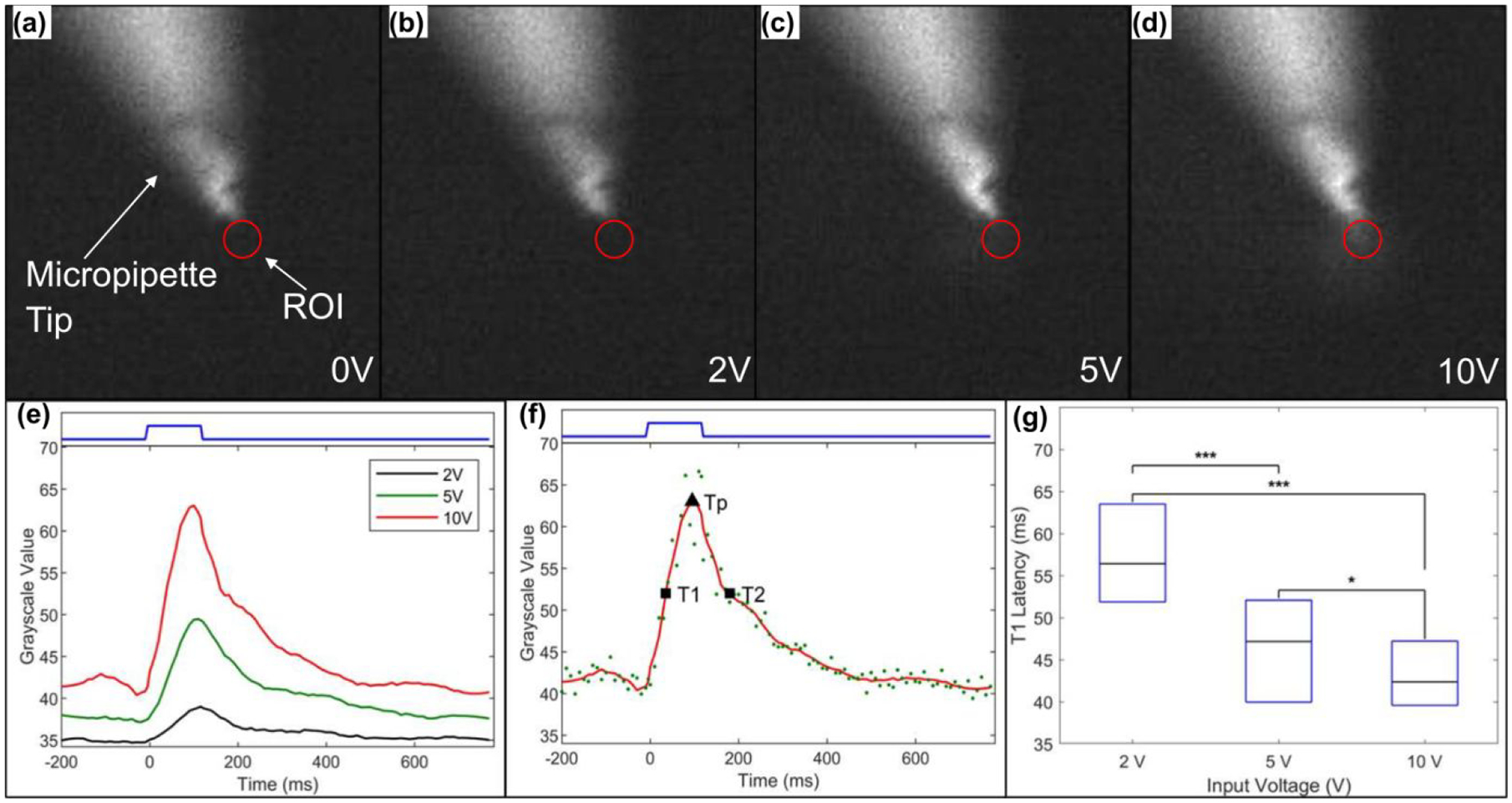
EOF onset and offset latency quantification using fluorescence measurement. (a) Frame showing the tip of the micropipette when no voltage was applied. A region of interest (ROI) was fixed near the micropipette outlet (red circle) with an area of 80 μm^2^ for measuring mean grayscale value (GV) in the region. (b-d) Maximum fluorescein bolus formation when 2, 5 and 10 V pulse was applied. (e) Change in GV within the ROI for a single representative pulse of 2, 5 and 10 V. (f) To capture the GV trend (green data points) the curve was smoothed using a Savitzky-Golay filter and latencies were expressed in terms of full width half maximum (FWHM) estimation. Y-intercepts (time to reach T1 and T2 when V is turned ON at t = 0s) on the filtered curve (red solid line) at half maximum are represented by square data points and peak is represented by a solid upright triangle (time duration Tp). (g) Comparison of boxplots containing lower quartile (bottom of box), upper quartile (top of box) and median (black line) of T1 latencies obtained from 2, 5, and 10 V injection pulses (n = 57 for each voltage value). A significant (p<0.001 denoted by ‘***’ and p<0.05 denoted by ‘*’) decrease in latency between voltage ON and T1 can be observed when voltage is increased from 2 V to 10 V. This indicates that lower glutamate injection onset (or lower T1 latency) can be achieved by using higher voltages.

It is essential for an EOF stimulator to initiate the fluid flow with minimal (or ideally no) latency. To measure the delay between the voltage command pulse and EOF onset we measured the intensity of the fluorescein emanating from the micropipette tip. A circular region of interest (ROI) with an area of 80 μm^2^ (red circle in Fig. 6a–d) was set adjacent to the micropipette outlet and mean grayscale value (GV) within the ROI was measured using image analysis software (Fiji, Schindelin et. al. [30]). These measured GVs were plotted against their respective capture time (green data points in Fig. 6f) and smoothed using Savitzky-Golay filtering (red line in Fig. 6f). The smoothed data were used to quantify EOF onset (switch ON) and offset (switch OFF) time by applying a full width at half maximum (FWHM) approach. Increasing the voltage amplitude produced higher mean GVs (Fig. 6e) and shorter T1 latencies (Fig. 6g). Fig. 6g also shows that EOF glutamate injections can support stimulation frequencies greater than 5 Hz for all three voltages. An EOF device thus should be able to inject glutamate pulses at a rate satisfactory for basic retinal stimulation [8].

### 3.3 Retinal responses to EOF driven glutamate

**Figure 7:**
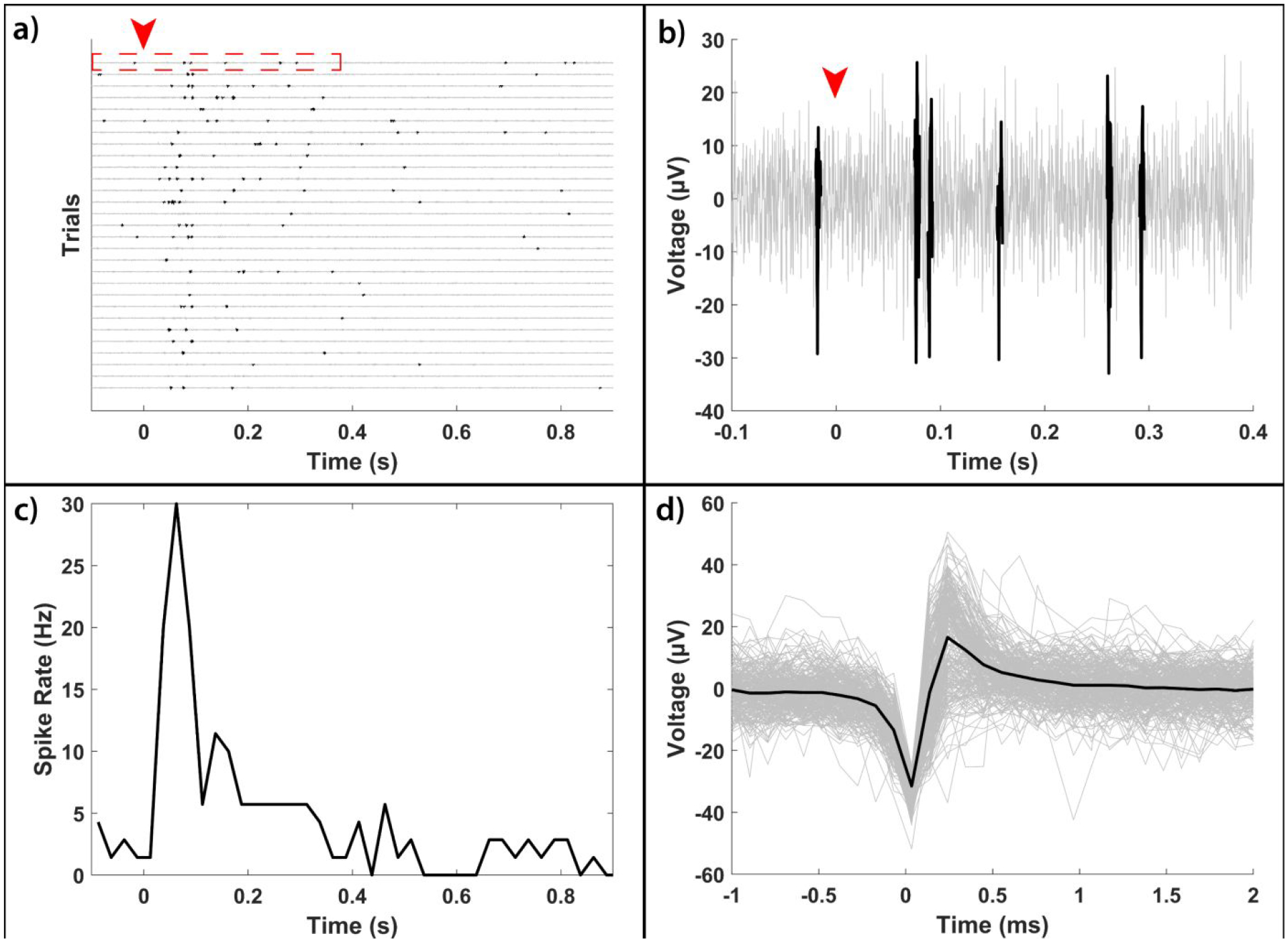
Representative plots obtained from an RGC when stimulated with 2V EOF-driven glutamate injections into the subretina of photoreceptor degenerated rats elicited purely chemical responses without electrical artifacts. a) A raw electrode recording from a channel with a responsive RGC with 200 Hz high pass hardware filtering. Each line shows the voltage trace from a single injection trial with detected spikes highlighted in black. As can be seen, there is an increase in activity approximately 50 ms after stimulation (time = 0 s) event shown by red downward arrow. b) A close up of the initial period of the last trial of subplot a) highlighted in red. EOF injections appear to cause exclusively chemically-evoked responses due to the lack of an electrical artefact, confirming the electrical isolation of the EOF injection device. c) A peristimulus time histogram (bin width of 25 ms) of the spikes highlighted in subplot (a) showing there is an excitatory response with a latency of approximately 50 ms. d) The extracted spike waveforms (gray) and the average spike waveform (black).

**Figure 8:**
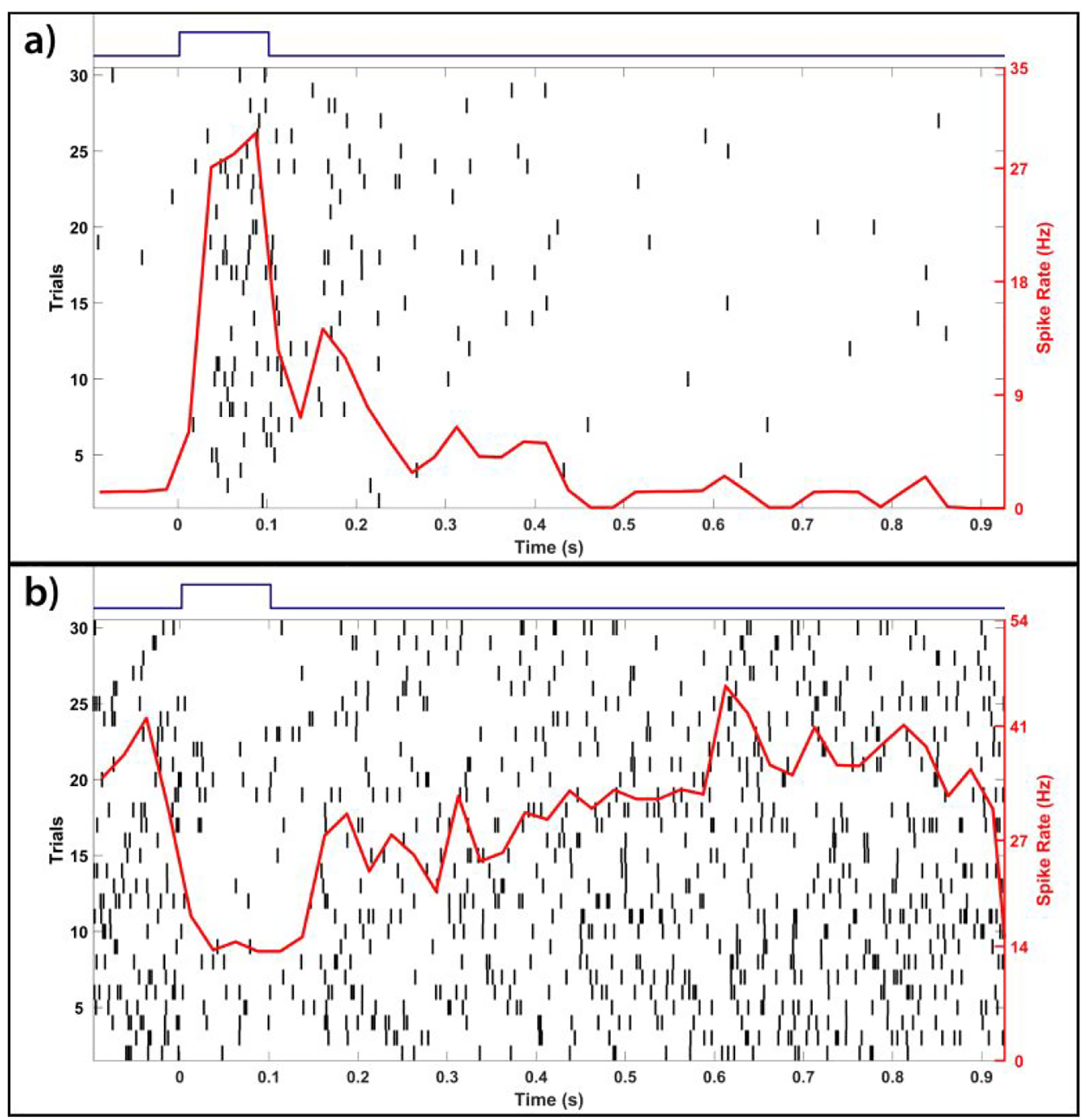
Electroosmotic flow (EOF) elicited excitatory (a) and inhibitory (b) spike rate responses. Subplots display representative spike rate and raster plots of two separate RGCs in response to 30 trials of 100 ms EOF injections of glutamate using a stimulus voltage of 2 V. Each vertical black line represents the timing of an individual spike with the trials stacked vertically (left y-axis) while the peristimulus time histogram (binwidth of 25 ms) of the spike rate is shown in red (right y-axis) averaged across all trials. The timing of each stimulus is represented by the blue square waves above each subplot.

To fully examine the potential for glutamate-evoked stimulation of the photoreceptor-degenerated retina using EOF, we conducted 92 sets of 100 ms pulse-width injections of 1 mM glutamate approximately 40 μm below the subretinal surface of 5 photoreceptor-degenerated retinas and studied a total of 55 glutamate-responsive RGCs. EOF-driven glutamate injections were found to elicit spike responses (Fig. 7a and b) exclusively through chemical stimulation since no electrical artifact was present in the multielectrode recordings (Fig. 7). The resulting RGC spike rate responses are demonstrated in Fig. 8, which shows the spike rate raster plots for two representative RGCs in response to a 2 V EOF glutamate injection. As can be seen, EOF injections evoked both excitatory and inhibitory spike rate responses where the majority (43 of 55 cells) of cells exhibited excitatory responses. Increasing voltages from 2V to 10V reduced the percentage of inhibitory responses from 27% (18/67 for 2V) to 20% (4/20 for 5V) and finally 12% (3/25 for 10V).

### 3.4 Spatial localization of EOF chemical stimulation

**Figure 9:**
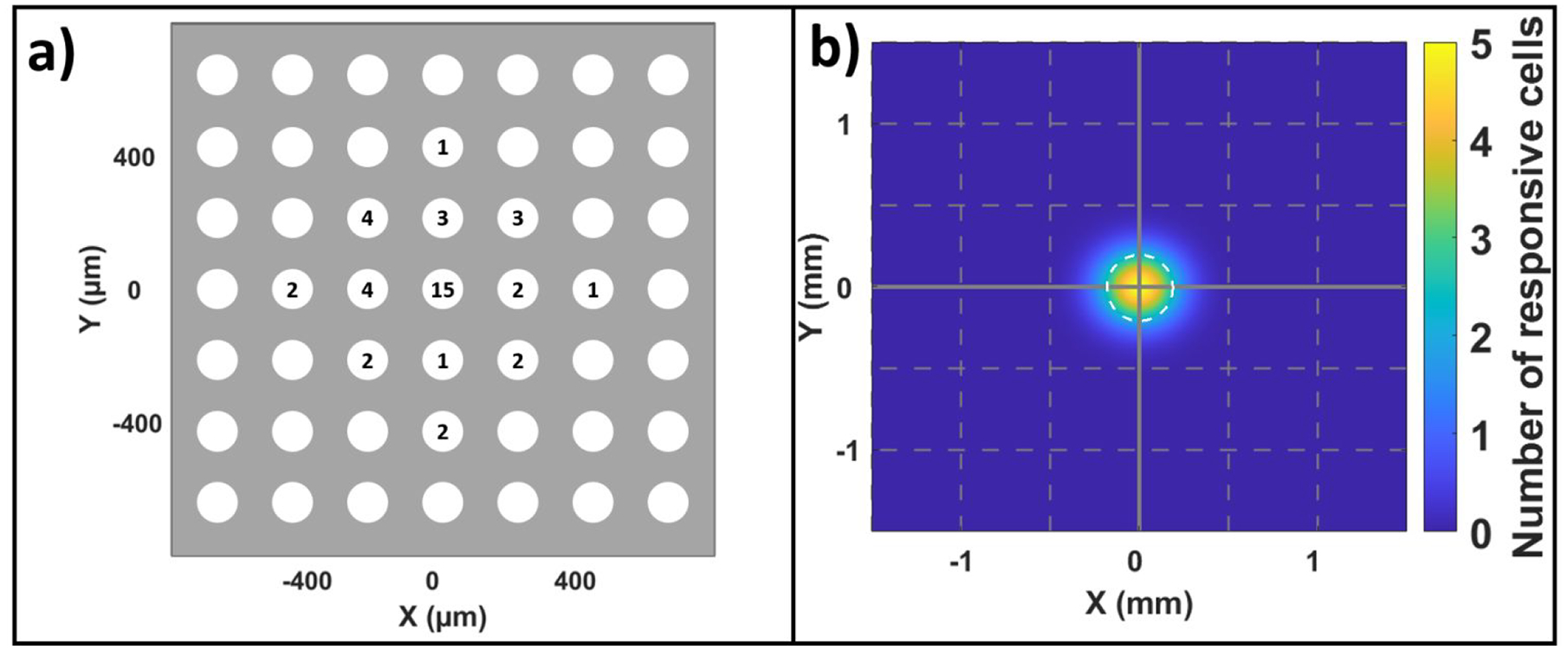
EOF-driven glutamate evokes spatially localized RGC responses. a) A two-dimensional layout of the pMEA electrodes (enlarged white circles) showing the number of responsive RGCs recorded by each electrode. A high proportion (approximately 36%) of all somal glutamate-responsive units were observed directly at the injection site (0,0) while the remaining 64% of responses were found on nearby electrodes. b) The spatial distribution of glutamate-responsive RGCs was fit with a two-dimensional Gaussian function with a median distance of 157 μm (value of w in the fit equation 1), which is shown by the white-dotted circle. The center of the plot represents the site of injection while the color indicates the density of RGC responses with warmer colors corresponding to higher densities. Somal, as opposed to axonal units, are shown since RGC axons can be located far from their respective cell bodies.

Because high spatial resolution is targeted for a biomimetic retinal prosthesis, we examined the spatial distribution of RGCs that responded to EOF-driven glutamate injections. As can be seen from 2 V EOF injections in Fig. 9a, a high percentage (approximately 36%) of the observed responsive somal cells were located at the site of injection (distance of 0 μm; center of plot) demonstrating the localization of EOF glutamate stimulation. Fig. 9b shows the spatial distribution of all glutamate-responsive RGCs relative to the injection site (center of plot) as a two-dimensional Gaussian spread function, which fit the data well (R^2 = 0.98). Warmer colors represent areas with higher densities of glutamate-responsive RGCs while the white-dotted circle shows the median distance of 157 μm measured with the Gaussian fit.

### 3.5 EOF-evoked glutamate responses are voltage dependent

**Figure 10:**
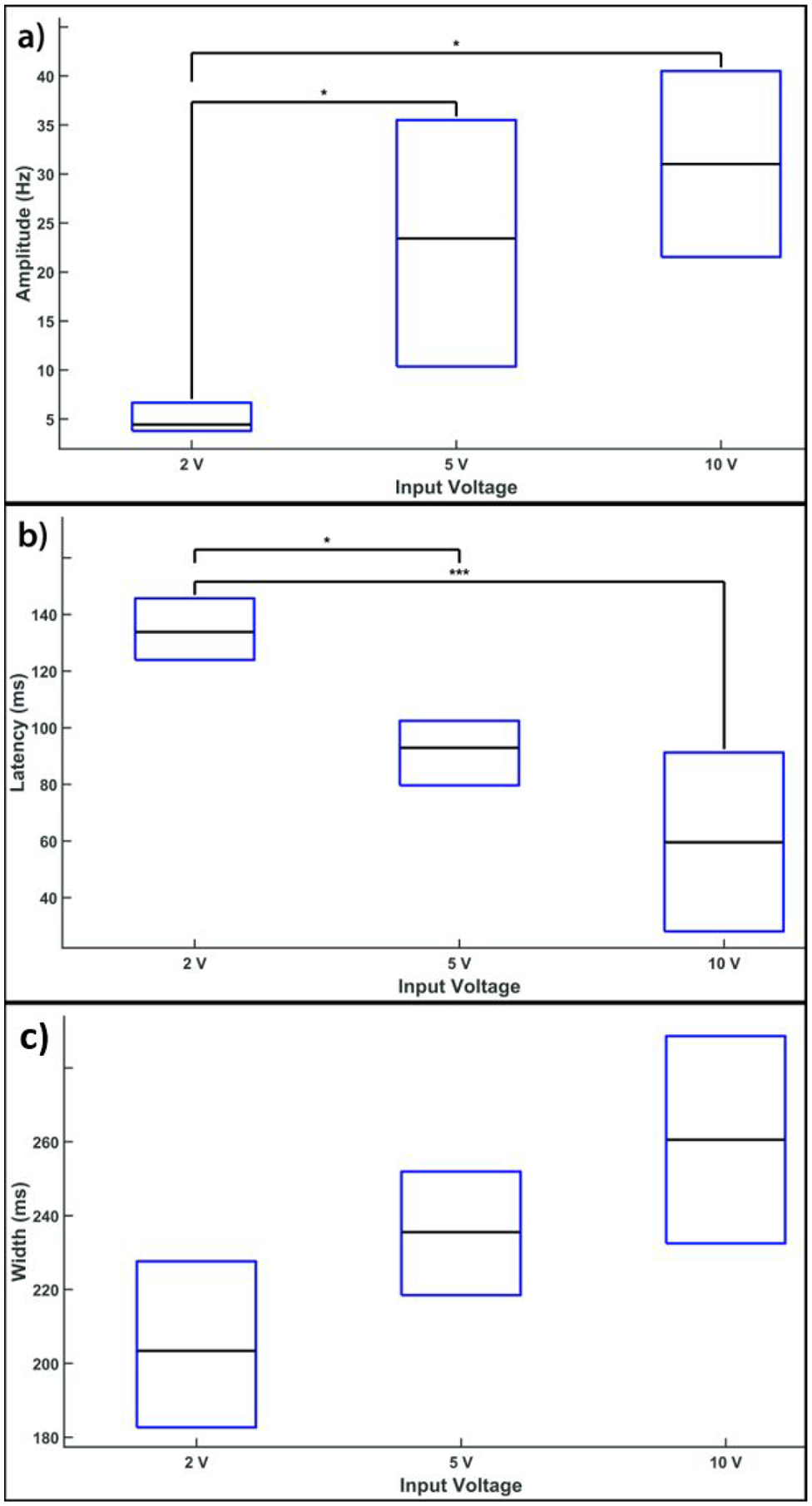
Glutamate-evoked excitatory spike rate responses vary based on the EOF voltage. (a) Comparison of the spike rate amplitude elicited by EOF glutamate stimulation showing the lower quartile (bottom of box), upper quartile (top of box), and median (black line in middle of box) of the amplitude for EOF voltages ranging from 2 to 10 V. The number of responses used for this analysis were 49, 16 and 22 for EOF injections at 2, 5 and 10 V respectively. As can be seen, increasing voltage above 2 V produced a significant (p<0.05 denoted by ‘*’on plot) increase in spike rate amplitude. (b) A similar comparison of the response latency (measured at the peak response) shows a significant reduction (p<0.001 denoted by ‘***’) in latency with high voltages (>2 V) compared with the minimum voltage of 2 V. (c) Increasing voltage caused an increase in the time-width of EOF-driven spike rate responses, although the increase did not reach statistical significance.

Since the pressure and velocity of EOF are dependent on the voltage applied, we explored a range of EOF voltages from 2 to 10 V to investigate how EOF voltage affected spike rate responses. Fig. 9 compares the spike rate amplitude (Fig. 10a), response latency (Fig. 10b), and response width (Fig. 10c) of glutamate-evoked spike rate responses. Higher EOF voltages (>2 V) were found to cause significantly larger amplitudes (p<0.05) and significantly faster responses (p<0.001 between 2 and 10 V and p<0.05 between 5 and 10 V). Increasing EOF voltages also had a seemingly real but statistically non-significant effect on the time width of responses, where higher voltages resulted in longer time widths (Fig. 10c).

## 4. Discussion

Chemical retinal stimulation seeks to elicit neural responses biomimetically by activating the cells responsible for vision using the neurotransmitter glutamate in a natural fashion [16]. Single site epiretinal and subretinal stimulation experiments [8,16,17], along with multisite patterned subretinal stimulation [18] have demonstrated that chemical stimulation offers substantial advantages. Subretinal chemical stimulation seeks to substitute for glutamate release characteristic of the lost photoreceptors (PRs) in a PR degenerated retina. Progressive morphological changes in the retina and neural connections are observed at various stages of PR degeneration [34–36], and subretinal chemical stimulation is found to be consistently effective at these various stages [37]. Chemical stimulation has the potential to provide high spatial resolution for artificial vision since the chemical injecting ports can be arranged in a dense array [18,38]. With electrical stimulation, however, there is risk of electrochemically induced tissue damage [14,39–41] as electrode packing increases and the stimulation electrode area is reduced. The above noted foundational studies in chemical retinal stimulation [8,16–18,37] used a commercially available external pressure injector to drive glutamate release pneumatically to evoke neural responses from retinal cells in-vitro. However, to transition chemical stimulation from in-vitro to in-vivo implementation, there is a need to develop a device with implant specific characteristics such as a microscale footprint, onboard power supply and an incorporated neurotransmitter actuation system. In this work, we have investigated the feasibility of using EOF as an actuation system to deliver small amounts of glutamate. We used commercially available components such as glass frits, micropipettes, microfluidic fittings and 3D printed parts to build a device to test EOF release of glutamate. Although envisaged by us as a means to control release of glutamate for a chemical retinal prosthesis, application of EOF as a microscale actuation mechanism for chemical release has far wider relevance. It could be used in many other chemical neural prostheses or for controlled drug delivery to various targets for situations in which the chemical to be injected is charged.

For an implantable chemical-based retinal prosthesis, there are certain design characteristics that suggested the use of EOF as the chemical driving mechanism in our work. EOF encourages fluid actuation through ports of a small diameter, which can be packed densely to permit high spatial resolution and to deliver low glutamate volumes [16], mitigating excitotoxicity due to overstimulation [42,43]. The ON-OFF switching time of EOF permits stimulation frequencies high enough to support visual tasks like reading. EOF achieves fluid propulsion without any moving parts or valves, which is critical for an implant to ensure longer working periods without maintenance. At the microscale, EOF can be generated with low voltages, which is an added advantage for an implant. Additionally, to ensure retinal stimulation exclusively due to the EOF by the neurotransmitter glutamate and not from electrical discharges from the electrodes (similar to electrical stimulation), direct contact between the electrodes and retinal cells can be avoided by insulating the electrodes from the retina.

An atypical design attribute of the mentioned EOF device is its electrode pair placement and elongated micropipette. By keeping the electrodes close together and away from the retina we were able to achieve stimulation at safe voltage levels. Furthermore, we grounded the cathode to prevent any conduction current between the electrodes and the retina through the conductive glutamate solution. As a result, electrical artifacts were absent and RGCs were stimulated purely through chemical means. These design attributes could be highly beneficial for systems oriented towards in-vivo neuromodulation and other nano or micro pipettors built for low volume handling. In the EOF device mentioned in this paper, the primary EOF driving element is a relatively large sintered glass frit (10 mm diameter, 2.5 mm thick, pore size between 4 and 5.5 μm) and required voltages of 2–10 V to actuate glutamate release. Although the parameters for diameter and thickness are much too large from an implant perspective, they served to establish the feasibility of chemical retinal stimulation using EOF as a viable propulsion mechanism. In our EOF device, the inter-electrode distance was 5 mm, resulting in an electric field of 400 V/m (or 0.4 mV/μm) for a 2 V actuation pulse. By reducing the inter-electrode distance using microfabricated microchannels and embedded electrodes one could decrease the voltage (and power) required for EOF actuation substantially.

EOF stimulation experiments conducted here yielded similar results for photoreceptor degenerated retinal stimulation compared to pneumatic stimulation in terms of the spike rate response characteristics and spatial localization. The excitatory spike rate amplitude, latency and response time width scaled as expected with different EOF actuation voltages. EOF injections had similar latencies (90 ms compared to 70 ms [17] and 75 ms [37]) to the prior work with pneumatic activation, and comparable spike rate amplitudes and time-widths. We postulate that the stimulation latencies with EOF injections were a combination of two phenomena: 1) latency between the input command signal and EOF onset from the micropipette tip (T1 latencies in the Characterization of EOF Switch Time section), and 2) the excitation latency of RGCs [7,8,18]. Although we have characterized the EOF system’s lag by using fluorescein injections in water, an in-depth investigation of flow characteristics in the micropipette-retina interface could provide further valuable insight in how to minimize in-vivo stimulation latencies. Based on the spike response latencies we expect our EOF device to be able to inject glutamate at a temporal rate of at least 5 Hz, comparable to the 6 Hz rate observed by Rountree et. al. [16]. Both studies were performed at room temperature. At physiological temperature, a higher temporal rate (comparable to those elicited for electrical retinal prostheses [44,45]) would be expected [46,47]. Nevertheless, the observed latencies in this work were well within the physiological range of response latencies reported for RGCs [48].

In terms of spatial localization, we were able to focus the glutamate injection using a fine micropipette tip (10 μm diameter) and evoke responses from a high number of cells (36% of somal units) directly at the injection site. At higher EOF actuation voltages of 5 V and 10 V, the number of responsive RGC somata at the injection site was lower (n = 8 units for each 5 V and 10 V) when compared to 2 V (n = 42). We do not have an explanation for this, though it is possible that a number of somal RGC spikes were masked by an increase in axonal spikes that is seen with higher voltage stimulation. Using RGC responses obtained from 2 V EOF injections, glutamate responsive somal units were well fit with a 2D Gaussian function that predicted a median distance of 157 μm. This is comparable to that obtained with pneumatic chemical stimulation where median glutamate spreads of 180 μm [16], 130 μm [17], 150 μm [37] and 400 μm [18] were observed. Our results also compare well with the recent clinically tested epiretinal implants, namely, epiretinal Argus II (315 μm) [49] and subretinal Alpha AMS (125 μm) [13,50], albeit, all are worse than the legal blindness limit. However, greater spatial localization has been achieved with in-vivo and in-vitro animal models using optogenetic (10 μm [51] and 63 μm [52]) and electrical (30 μm [53]) stimulation. For us to attain as localized stimulation with glutamate will likely require dispensing the neurotransmitter in less than the 1 mM concentration used here.

Similar to other stimulation strategies (namely – ultrasonic [54], optogenetic [55] and electrical [44,56,57]), EOF injections were also observed to elicit excitatory and inhibitory responses, however the majority (73 %, 80% and 88% for 2, 5 and 10 V respectively) of RGC responses were excitatory which is similar to earlier pneumatic glutamate injection studies that reported an excitatory RGC population of 70.4 % [16] and 80 % [37]. Although the specific mechanisms for glutamate elicited excitatory and inhibitory responses have yet to be determined, we believe they must include stimulation of cells of the retina’s inner nuclear layer, since RGCs themselves display only excitatory responses to glutamate. Further work is needed to ascertain the precise cellular origins of the differential responses.

Another issue that needs to be addressed is the dose of glutamate to dispense. Here we used nano liter doses of 1 mM glutamate to replicate that used in a previous study with pneumatic pressure injection [8] so that our results were directly comparable to results from that study. Diffusion and extraction of glutamate from the in-vivo retina will likely be quite different from the situation in the in-vitro preparation used here. We currently envision an implantable device in which the dispensing outlets will be pushed 40 μm into the retina from the subretinal side. Key questions that will need to be asked are (1) what dose of glutamate is needed to evoke physiological responses from retinal ganglion cells, and (2) does such a dose cause any excitotoxicity [42], particularly in a retina with photoreceptors lost. The 1 mM concentration used here is a good starting point but the optimal dose remains to be determined.

Unlike the single point stimulation approach used in this study, a microfabricated device with multiple, independently addressable, injection ports would be required to generate an image pattern on the retina. Multisite chemical stimulation can produce activation through summation of otherwise subthreshold quantities of neurotransmitter coming from neighboring ports and converging through diffusion to a common point [18]. A similar phenomenon of subthreshold summation occurs with electrical stimulation and one supposes that the field of chemical retinal prostheses can benefit from the lessons of multisite stimulation learnt earlier for electrical retinal prostheses.

The first step towards such a device would be to determine suitable biocompatible materials [58] that can be microfabricated in a form that is multilayered, implantable within the subretinal space, and flexibly conforms to the natural curvature of the retina [59,60]. The microfabrication strategy should also consider integration of the various layers of the device (see Fig.1 in the Introduction Section) that includes an onboard fluidic reservoir for glutamate, porous layer, integrated electrodes across individual pores to generate EOF injections, and hollow microneedles to penetrate the glial seal [61] formed in the photodegenerated retina and deliver boluses of glutamate near the synaptic junctions of cells of the inner nuclear layer (INL). Chronic implantation studies must be conducted to assess the bioactivity of stored glutamate and biostability of such an EOF powered chemical retinal prosthesis. Additionally, retinal phantoms [62,63] could be employed for optimizing surgical processes.

## Conclusion

Chemical retinal stimulation is a promising biomimetic method of engaging retinal neurons and, in this paper, we have demonstrated the feasibility of using EOF to drive exogenous glutamate to elicit neural responses in rat retinae in-vitro. By comparing with results obtained from previous chemical stimulation experiments that used an external pneumatic pressure injector to drive glutamate, we have demonstrated that the EOF technique can achieve comparable performance in a more compact package that can be miniaturized substantially using current microfabrication techniques. Flow rate and switching time characterization of the EOF device indicates controllable glutamate injections (in nanoliters per second) can be delivered at low voltages, enabling a future chemical prosthesis to work with a limited energy budget. Satisfactory glutamate induced stimulation of retinal cells with localized responses was observed. Response amplitude scales with the driving voltage. This work presents a course towards realizing an implantable chemical retinal prosthesis that can restore vision in patients suffering from photoreceptor degenerative diseases.
